# SubMachine: Web‐Based Tools for Exploring Seismic Tomography and Other Models of Earth's Deep Interior

**DOI:** 10.1029/2018GC007431

**Published:** 2018-05-11

**Authors:** Kasra Hosseini, Kara J. Matthews, Karin Sigloch, Grace E. Shephard, Mathew Domeier, Maria Tsekhmistrenko

**Affiliations:** ^1^ Department of Earth Sciences University of Oxford Oxford United Kingdom; ^2^ Centre for Earth Evolution and Dynamics, Department of Geosciences University of Oslo Oslo Norway

**Keywords:** data visualization, seismic tomography, deep Earth, mantle, subduction, geological and geophysical data sets

## Abstract

We present SubMachine, a collection of web‐based tools for the interactive visualization, analysis, and quantitative comparison of global‐scale data sets of the Earth's interior. SubMachine focuses on making regional and global‐scale seismic tomography models easily accessible to the wider solid Earth community, in order to facilitate collaborative exploration. We have written software tools to visualize and explore over 30 tomography models—individually, side‐by‐side, or through statistical and averaging tools. SubMachine also serves various nontomographic data sets that are pertinent to the interpretation of mantle structure and complement the tomographies. These include plate reconstruction models, normal mode observations, global crustal structure, shear wave splitting, as well as geoid, marine gravity, vertical gravity gradients, and global topography in adjustable degrees of spherical harmonic resolution. By providing repository infrastructure, SubMachine encourages and supports community contributions via submission of data sets or feedback on the implemented toolkits.

## Key Points

Web‐based tools for the interactive visualization, analysis, and quantitative comparison of global‐scale, volumetric (3‐D) data sets of the subsurface
Focus on global seismic tomography models from body waves, surface waves, and normal modes (>30 models currently implemented)
Additional tools for interacting with related data sets: plate tectonic reconstructions, topography, geoid, marine gravity, normal mode observations, etc

## 1 Introduction

Seismic tomography is a powerful geophysical imaging method that has been yielding increasingly detailed structural information about the Earth's deep interior. Applied on a planetary scale, it uses seismic waves, generated by tens to thousands of moderate to large earthquakes, to sample and estimate the 3‐D spatial distribution of heterogeneities in the crust and mantle. Such heterogeneities cause seismic waves to propagate at slightly faster or slower velocities than average ambient mantle or crust, the structure of which is reasonably well known (Dziewonski & Anderson, 1981; Kennett & Engdahl, 1991; Kennett et al., 1995). Although seismic velocity anomalies (dv/v) are of secondary interest per se, they correlate with density, temperature, and compositional anomalies, which are the drivers of heat and material flows in the solid Earth.

Due to the vast amounts of data and the heavy computational demands, generating a whole‐mantle tomography model is a major, nonroutine effort. Each new model tends to be parameterized and published in a different format, partly reflecting the rapidly evolving nature of tomography, and partly the lack of community effort to standardize outputs. It is often not straightforward to explore a seismic tomography model without specialized knowledge and a customized programing effort. Hence, these models more often exist as the limited number of 2‐D slice plots seen in original publications rather than as full, 3‐D data sets that are readily accessed and reinspected. Much of the information they contain is never unlocked, which limits their application toward understanding Earth structure and evolution. SubMachine's main purpose is to make the solid Earth community more productive by making seismic tomography models easily accessible to further exploration.

Seismic tomography is a mature and robust technique. Since its inception (Aki et al., 1977; Aki & Lee, 1976), its imaging results have steadily converged in some contexts, in that newer models tend to be more sharply resolved versions of older ones using the same types of wave data. Furthermore, models computed from different wave types (i.e., body waves, surface waves, or normal models) tend to be consistent after accounting for their various artifacts, which afflict any underdetermined inverse problem, but often in predictable ways. This convergence of results motivates the comparison of tomography models quantitatively and in detail, as SubMachine enables its users to perform. Mantle convection modeling or magnetotelluric studies produce lithospheric to planetary‐scale mantle models that would be equally suited to visualization in SubMachine, but we have not prioritized them for this first release, in part because they do not yet show similar degrees of convergence.

**Figure 1:**
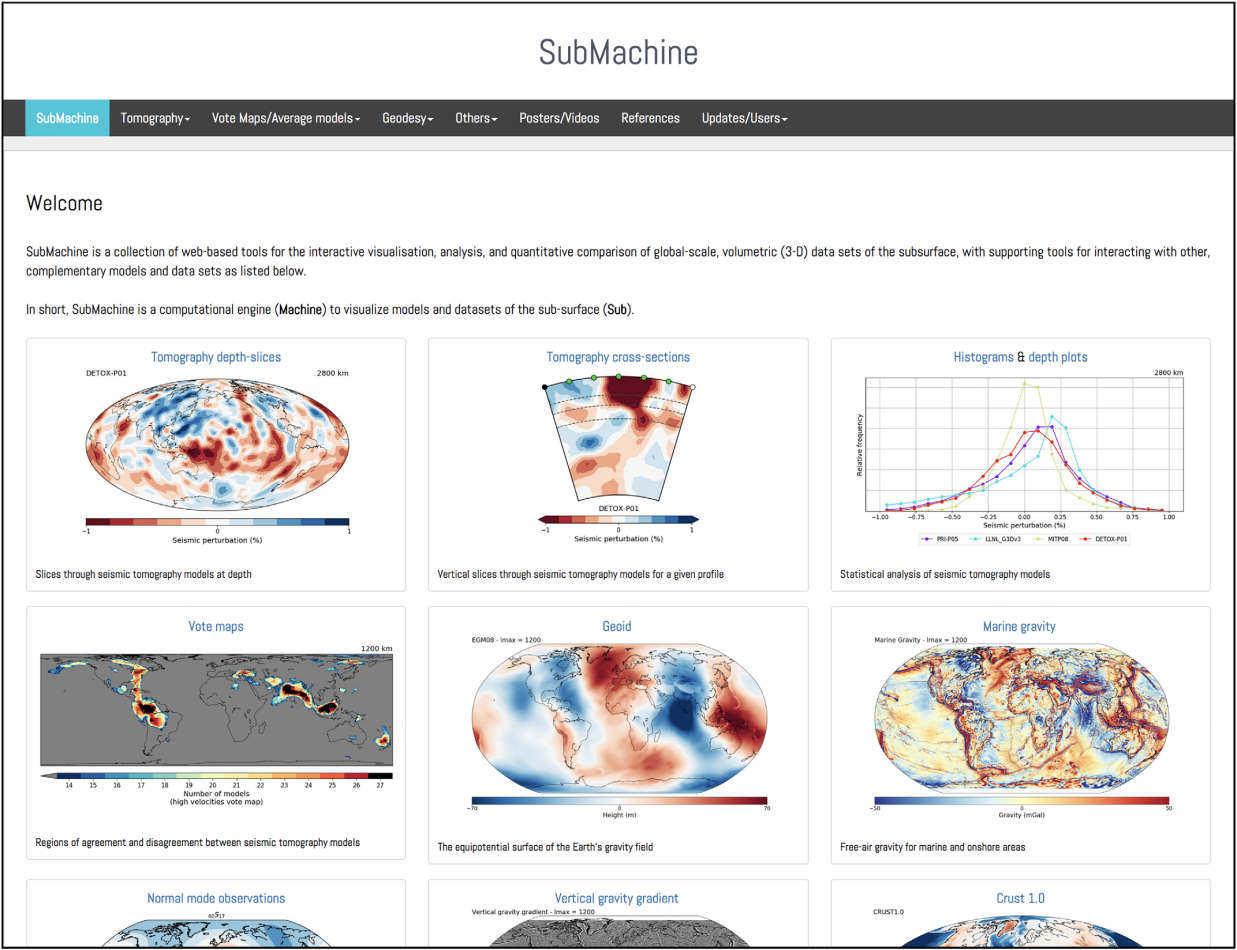
Screenshot of the home page of the SubMachine web portal (http://submachine.earth.ox.ac.uk). The first release includes functionalities for the visualization, analysis and quantitative comparison of over 30 global and regional seismic tomography models, as well as complementary data sets that are pertinent to interpreting mantle structure and evolution.

The full, volumetric parameter data sets of seismic tomography models can usually be obtained freely from published online supplements, from the Earth Model Collaboration (EMC) website (http://ds.iris.edu/ds/products/emc/) of the Incorporated Research Institutions for Seismology (IRIS), or by contacting their authors. For the SubMachine portal, we have assembled more than 30 global body wave, surface‐wave, and normal mode models and have processed them into a common format. In this first release of the SubMachine web portal, software tools to visualize and explore the models—individually, side‐by‐side, or through statistics and averaging tools—are provided. The appearance of SubMachine's home page is shown in Figure 1.

Compilations of tomographic models and basic plotting tools have been made available by some organizations and research groups, but SubMachine provides a more expansive toolkit for visualization and analysis of such models as well as complimentary (nontomographic), global‐scale Earth data sets. The implementation of the IRIS EMC in 2011 was a major step toward the collection, homogenization, and dissemination of seismological processing outputs, and this community‐supported repository of mainly tomography models has introduced a uniform model and metadata format (Trabant et al., 2012). Its web‐based visualization tools support the generation of horizontal slices, vertical slices, and velocity‐depth profiles through 3‐D models, and download of the raw numbers (dv/v values) visualized in the tomogram slices as ASCII or netCDF files. As of December 2017, EMC serves 49 tomography models, many of them regional in scale or updates of older models; its collection of 21 global tomography models is a subset of those supported in SubMachine, with a few exceptions. Unlike SubMachine, EMC's visualization works best for regional‐scale mantle models (no spherical rendering), and there is no support for the direct comparison of two or more models. IRIS EMC does not serve the complementary data sets that SubMachine supports, such as marine gravity, vertical gravity gradient, geoid, global topography, global crustal structure, normal mode observations, vote maps, and time‐dependent plate reconstructions.

A web‐based visualization effort focused on global tomography is CSMAP at JAMSTEC (http://csmap.jamstec.go.jp/csmap/ [last accessed: December 2017]; Kim et al., 2016). As of December 2017, vertical sections can be extracted from five *P* wave and *S* wave models. There is no support for horizontal sections, download of raw dv/v values, or nontomographic data sets.

In the preportal era, the tomographic model comparison by Becker and Boschi (2002) (http://www-udc.ig.utexas.edu/external/becker/tomography/ [last accessed: March 2018]) has been influential as a pioneering effort in serving the community with a collection of global tomographic models in a homogenized format, on a well‐maintained website, and with basic visualization scripts.

Nontomographic data sets play a supporting role in the conception of SubMachine. They are the focus of other sophisticated, web‐based community portals such as GeoMapApp (http://www.geomapapp.org/), the OneGeology Portal (http://portal.onegeology.org/OnegeologyGlobal/), and the GPlates Portal (http://portal.gplates.org/; Müller et al., 2016) [all last accessed: December 2017].

Section 2 explains SubMachine's architecture and its functionalities: visualization of global tomography models, i.e., volumetric data sets, and related statistics (section 2.1), and the comparison of models through tomographic “vote maps” which are generated by applying a one‐bit (binary digit) thresholding operation to two or more tomography models and then stacking them (Shephard et al., 2017; section 2.2). Section 3 explains the visualization of static and time‐dependent observations and models that are pertinent to the interpretation of mantle structure and are complementary to seismic tomography. This currently include models of the geoid (Pavlis et al., 2012), free‐air gravity (Sandwell et al., 2014), vertical gravity gradient (Sandwell et al., 2014), and topography and bathymetry (Olson et al., 2014), all in adjustable spherical harmonic degrees. Also included are global crustal model CRUST1.0 (Laske et al., 2013), normal mode observations and their sensitivities for a selection of upper mantle and lower mantle modes (Koelemeijer, 2014; Koelemeijer et al., 2013), and a suite of time‐dependent plate reconstruction models (Matthews et al., 2016; Seton et al., 2012; Zahirovic et al., 2016). Section 4 raises discussion points about SubMachine's current performance and future directions.

## 2 SubMachine Architecture and Functionality

The SubMachine portal is based on a three‐tiered client‐server architecture, comprising a data layer (database of tomography models and other data), a logical layer (processing and calculations), and a presentation layer (graphical user interface [GUI]). The user interface, written in HTML, PHP, and JavaScript collects user inputs and sends them to the logical layer, which creates variables based on the user inputs and passes them to the visualization and statistical analysis tools. The codes of the logical layer, written mainly in Python and PHP, interact with the data layer to extract slices or other subsets of the volumetric and surface data sets, and to generate and store the plots and other outputs.

SubMachine's current data holdings take up ∼20 GB of storage on a server at the University of Oxford. Tomography models are data sets in three spatial dimensions, as are tomography vote maps, which are one‐bit thresholded stacks of several tomography models. Surface data sets are in two (horizontal) spatial dimensions, e.g., plate reconstructions, geoid, gravity, or topography. Data sets can have an additional time property, e.g., plate reconstructions evolving over geologic time. By defining a mapping function, time‐dependent data sets can be linked to tomography, for example, by mapping geologic time to mantle depth when considering the sinking rates of subducted slabs. Thus, plate reconstruction models or hotspot locations can be combined with tomography models or vote maps to produce spatiotemporal comparisons between surface dynamics and the Earth's interior structure.

SubMachine puts an emphasis on facilitating user‐defined model comparisons. Tomography models can be homogenized for display against the same reference Earth models. Multiple 3‐D and 2‐D data sets can be plotted with the same map projections and coloring schemes. Vote maps using adjustable voting criteria are another comparison tool, as are tools to compute and plot model statistics.

Each of the subsections that follow discusses one webpage (“tab”) of the SubMachine portal (http://submachine.earth.ox.ac.uk). These tabs are tomography “Depth slices,” “Cross sections,” “Velocity histograms,” and “Velocity‐Depth profiles” (section 2.1), tomography “Vote Maps” (section 2.2), tectonic plate reconstructions (section 3.1), “Geodesy” (section 3.2), global crustal structure “Crust 1.0” (section 3.3), and “Normal Modes” observations (section 3.4).

### 2.1 Tomography Visualization

SubMachine's “Tomography” tab currently supports the visualization of 36 tomography models. 32 are global, whole‐mantle models, two are global, upper mantle models, one is a global, midmantle model, and one is a regional mantle model (from the surface down to 1,800 km depth) for North America. Table 1 details the model sources and original references. This information also displays at the bottom of the webpage. Models were obtained from IRIS EMC (Hutko et al., 2017), from private or institutional websites associated with the authors, from published supplementary materials, or by personal communication with the authors.

**Table 1:** Tomography P Wave and S Wave Models Currently Supported in SubMachine

Model*	Data type	Reference model	Reference	Source
DETOX‐P01	Body waves	IASP91	Hosseini and Sigloch ( 2015) Hosseini ( 2016)	From lead author of this study
GAP‐P4	Body waves	GAP	Fukao and Obayashi ( 2013) Obayashi et al. ( 2013)	JAMSTEC Data Catalog website
GyPSuM‐P	Body waves	PREM	Simmons et al. ( 2010)	IRIS EMC website
HMSL‐P06	Surface waves, body waves	AK135	Houser et al. ( 2008)	Personal website (Christine Houser)
LLNL_G3Dv3	Body waves	Custom averaged model	Simmons et al. ( 2012)	Lawrence Livermore National Laboratory (LLNL) website
MITP08	Body waves	AK135	Li et al. ( 2008)	Published supplementary material
MITP_USA_2011MAR	Body waves	AK135	Burdick et al. ( 2012)	Published supplementary material
MITP_USA_2016MAY	Body waves	AK135	Burdick et al. ( 2017)	Published supplementary material
PMEAN	Averaging tomography models	PREM	Becker and Boschi ( 2002)	Personal website (Thorsten Becker)
PRI‐P05	Body waves	IASP91	Montelli et al. ( 2006)	GLOBALSEIS website
Sigloch_NAm_2011^a^	Body waves	IASP91	Sigloch ( 2011)	Published supplementary material
SP12RTS‐P	Surface waves, body waves, normal modes	PREM	Koelemeijer et al. ( 2016)	Personal website (Paula Koelemeijer)
SPani‐P	Surface waves, body waves	PREM	Tesoniero et al. ( 2015)	Personal GitHub page (Andrea Tesoniero)
UU‐P07	Body waves	AK135	Amaru ( 2007)	Personal communication (Wim Spakman)
3D2016_09Sv^b^	Surface waves	Custom averaged model	Debayle et al. ( 2016)	Personal website (Eric Debayle)
GyPSuM‐S	Body waves	TNA/SNA	Simmons et al. ( 2010)	IRIS EMC website
HMSL‐S06	Surface waves, body waves	AK135	Houser et al. ( 2008)	Personal website (Christine Houser)
PRI‐S05	Body waves	IASP91	Montelli et al. ( 2006)	GLOBALSEIS website
S10MEAN	Averaging 10 tomography models	None	Doubrovine et al. ( 2016)	Personal communication (Pavel Doubrovine)
S20RTS	Surface waves, body waves, normal modes	PREM	Ritsema et al. ( 1999)	Personal website (Paula Koelemeijer)
S362ANI+M	Surface waves, body waves, normal modes	STW105	Moulik and Ekström ( 2014)	Personal website (Raj Moulik)
S40RTS	Surface waves, body waves, normal modes	PREM	Ritsema et al. ( 2011)	Personal website (Paula Koelemeijer)
SAVANI	Surface waves, body waves	PREM	Auer et al. ( 2014)	Personal website (Thorsten Becker)
SAW642ANb	Waveform	PREM	Panning et al. ( 2010)	Personal website (Mark Panning)
SEISGLOB1	Surface waves, normal modes	PREM	Durand et al. ( 2016)	IRIS EMC website
SEISGLOB2	Surface waves, body waves, normal modes	PREM	Durand et al. ( 2017)	IRIS EMC website
SEMUCB‐WM1	Waveform	Custom averaged model	French and Romanowicz ( 2014)	Seismo Berkeley website
SEMum	Waveform	PREM	Lekić and Romanowicz ( 2011)	IRIS EMC website
SGLOBE‐rani	Surface waves, body waves	PREM	Chang et al. ( 2015)	IRIS EMC website
SL2013sv^b^	Surface waves	AK135	Schaeffer and Lebedev ( 2013)	Personal website (Andrew Schaeffer)
SMEAN	Averaging tomography models	PREM	Becker and Boschi ( 2002)	Personal website (Thorsten Becker)
SP12RTS‐S	Surface waves, body waves, normal modes	PREM	Koelemeijer et al. ( 2016)	Personal website (Paula Koelemeijer)
SPani‐S	Surface waves, body waves	PREM	Tesoniero et al. ( 2015)	Personal GitHub page (Andrea Tesoniero)
TX2011	Body waves	TX2011_ref	Grand ( 2002)	IRIS EMC website
TX2015	Body waves	TX2011_ref	Lu and Grand ( 2016)	Personal communication (Stephen Grand)
Zaroli2016^c^	Body waves	IASP91	Zaroli ( 2016)	Personal communication (Christophe Zaroli)

*Note. S* wave models are shown in the grey‐shaded rows.

a Regional mantle model for North America.

b Upper mantle model.

c Global midmantle model.

The 36 models were accessed in many different original parameterizations. Horizontally, these can be regular or irregular localized grids, or spherical harmonic basis functions; in the third dimension, regular or irregular depth layers, possibly interpolated by spline functions. We have linearly interpolated each model on a regular horizontal grid of 0.5° × 0.5° using Generic Mapping Tools (GMT, version 5.3.1; Wessel et al., 2013). The highest lateral resolution in the original model parameterizations was 1° × 1°, hence, interpolation on a grid of 0.5° × 0.5° retains all original information and introduces no aliasing error, while keeping the computational effort for processing and plotting acceptable.

In the vertical dimension, we retain the exact, discrete depth layers specified by the original model parameterizations. This assures that all the depth‐dependent complexities in tomography models are preserved, such as velocity changes in the lithosphere‐asthenosphere boundary, transition zone, and core‐mantle boundary regions. In case of irregular parameterization in depth (e.g., tetrahedral volume mesh), our interpolation extracted uniform depth increments of 50 km. Moreover, for each discontinuity in the background model, two depths closely bracketing that discontinuity were extracted and stored in the data set, e.g., depth slices at 650 and 670 km depth in case of a 660 km discontinuity. If a user requests data that do not coincide with points on this fine, precomputed grid, SubMachine interpolates linearly on the fly.

**Figure 2:**
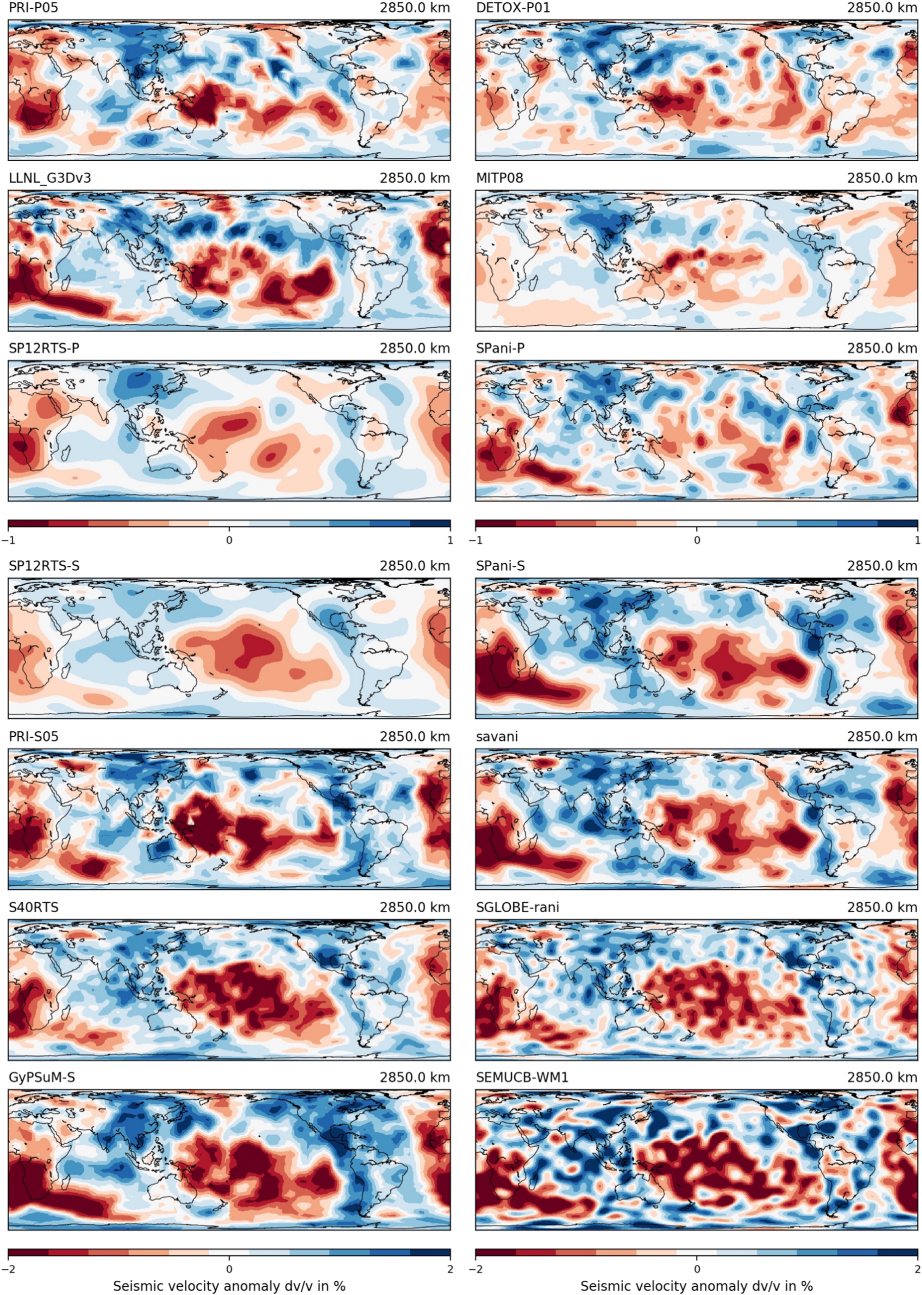
“Tomography‐Depth slices” functionality. Figure shows SubMachine's plotting output for a user‐defined comparison of 3‐D global‐scale tomography models at a specified depth, in this case six *P* wave and eight *S* wave models at 2,850 km. Model names are shown above each figure, see Table 1 for model descriptions and references. The map projection is user defined, here cylindrical equal area. Color denotes seismic velocity anomalies dv/v relative to a spherically symmetric reference velocity model. A variety of color bars are implemented in addition to the classic red‐blue, discretized scheme shown here. The models become comparable by subtracting each model's spatially averaged dv/v anomaly at the selected depth. It is also possible to plot the models relative to a common, user‐specified reference model, an option not used here. Table 1, column 3 lists the original reference models for each tomography models. Longitude, latitude and dv/v values (in %) contained in each 2‐D map slice can be downloaded in ASCII format using the “Download grid” option.

**Figure 3:**
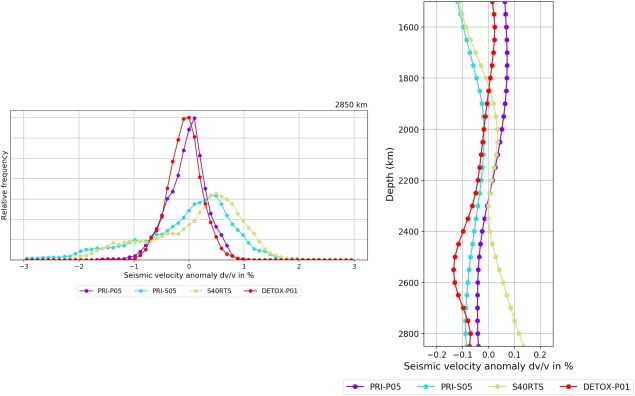
Output of SubMachine's “Tomography‐Velocity histograms” and “Tomography‐Velocity‐Depth profiles” functionalities. (left) Velocity histograms show the relative frequency of dv/v anomalies at a user‐specified depth, in this case 2,850 km, in a comparison of four global models. (right) Velocity‐depth profiles show the mean of velocity anomalies at user‐defined depths (minimum, maximum and interval). Velocity histograms and velocity‐depth profiles can be computed relative to the same reference Earth model and/or each model's mean dv/v at each depth can be removed (options not selected here). This example shows observational evidence for “Large Low Shear Velocity Provinces” (LLSVPs) in the lowermost mantle (Masters et al., 2000; Megnin & Romanowicz, 2000; Montelli et al., 2006; Ritsema & van Heijst, 2002; Woodhouse & Dziewonski, 1989): histograms of shear wave models (PRI‐S05 and S40RTS) have long and heavy tails of negative dvs/vs, indicating regions that are much slower than average. The positive half of the S‐model histograms shows no heavy tails (despite the mode of the distribution falling in the positive dv/v range). Histograms of *P* wave models show no significant asymmetry, in particular no heavy tails of negative dvp/vp. (Such spatial averaging obscures the observation that extremely slow dvs/vs are localized in two large areas of the southern hemisphere, the LLSVPs.)

**Figure 4:**
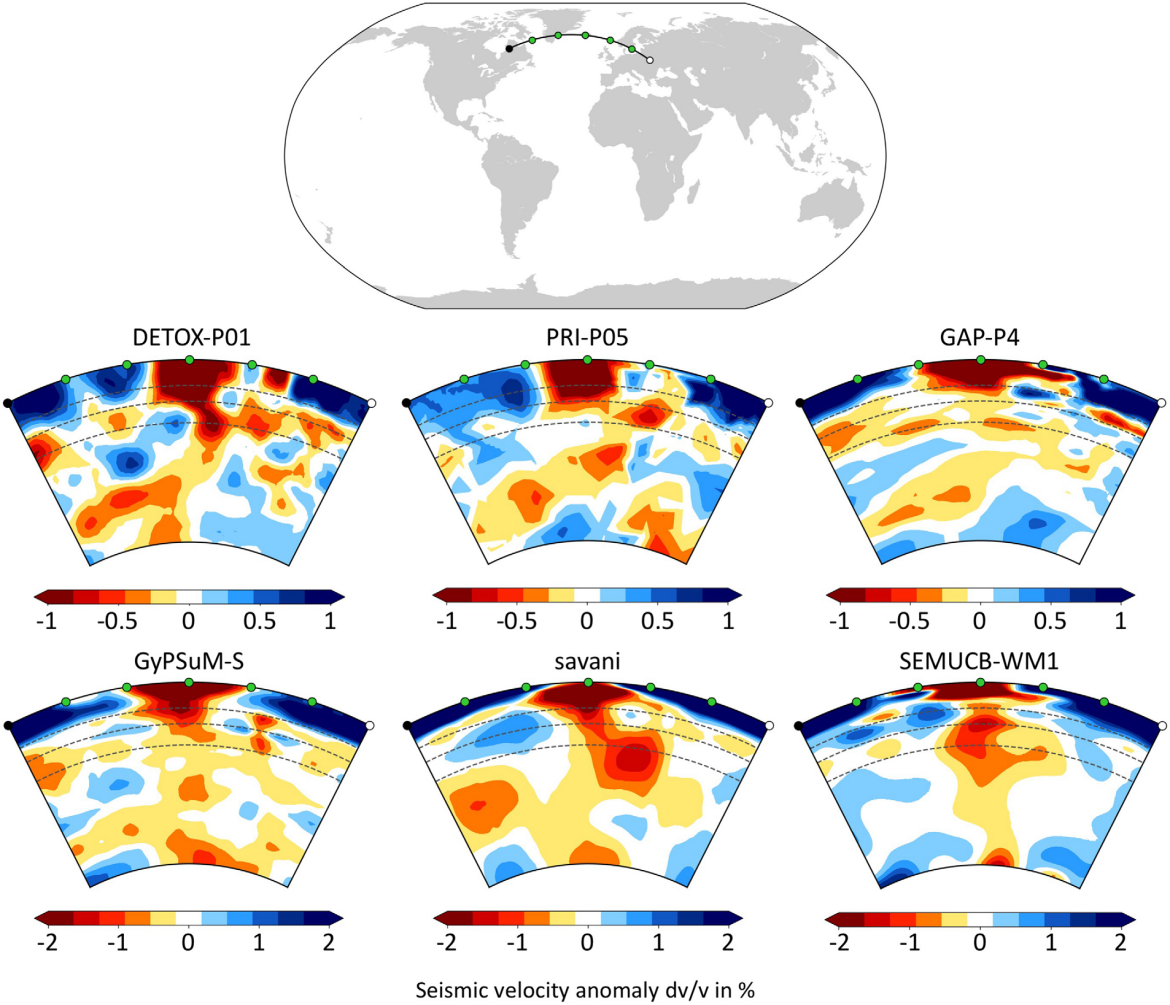
Plotting output of SubMachine's “Tomography‐Cross sections” functionality. Example shows a comparison of six selected global tomography models for a section through the North Atlantic and Iceland, from the surface to the core‐mantle boundary. *“*410,” “660,” and “1,000” km depths are shown as dashed lines. This example demonstrates how, within the space of minutes, a user can get a sense for the state of the art in the imaging of mantle plumes. A cross section can be specified either by its two end points, or by its middle point + azimuth + length. SubMachine uses Python Visualization Toolkit libraries for efficient reading and slicing of volumetric data sets. x, y, z, and dv/v values (in %) contained in each cross section can be downloaded in ASCII format using “Download grid” option.

The “Tomography” tab includes subpages for “Depth slices,” “Cross sections,” “Velocity histograms,” and “Velocity‐Depth profiles,” the functionalities and outputs of which are illustrated in Figures 2, 3, 4. Depth slices are horizontal 2‐D sections (map sections) through a volumetric model at a fixed depth (Figure 2); velocity histograms are summary representations of the velocity anomalies dv/v contained in such a depth slice (Figure 3). Velocity‐depth profiles are computed from many horizontal slices at different depths, i.e., from 3‐D data. On each horizontal slice, a selected statistical parameter (e.g., mean, root mean square [rms], standard deviation [std]) is calculated, and this parameter is presented in a 2‐D plot, as a function of depth (Figure 3). Cross sections are vertical 2‐D sections along a great circle path at the Earth's surface and through its centre (Figure 4). To define the arc length of a section, the user must either specify the latitudes and longitudes of two end points at the surface or the latitude and longitude of a midpoint, along with an azimuth and distance (in km).

Figure 2 shows output of SubMachine's “Depth slices” functionality, for a slice at 2,850 km depth through 14 *P* wave and *S* wave tomography models. Plotting two or more models side‐by‐side facilitates model comparison and currently sets SubMachine apart from other online visualization tools. This comparison reveals for example overwhelming agreement on two large‐scale, low‐velocity structures beneath the Pacific and Africa (the so‐called Large Low Shear Velocity Provinces; LLSVPs), and on fast‐velocity anomalies beneath Eastern Asia and the Americas.

The user can default to viewing all tomography models in their originally published forms, i.e., relative to the spherical reference Earth models used in their generation. Alternatively, models can be homogenized and displayed relative to a single reference model, one of “PREM,” “IASP91,” or “AK135” (Dziewonski & Anderson, 1981; Kennett & Engdahl, 1991; Kennett et al., 1995). In case of an anisotropic background model (e.g., PREM between 80 and 220 km depth), the isotropic (Voigt) average wave speed is computed. Another homogenization option is to remove the mean dv/v values from depth slices and histograms. We note that one common regularization choice in seismic tomography is norm damping, which penalizes large (positive or negative) dv/v deviations from the reference model. Models produced without norm damping are thus more accepting of bias from the reference model, and even where norm damping is used, it is usually applied globally to the entire mantle volume, so that individual depth slices may still be biased from the reference model (Nolet, 2008). Retrospective removal of the mean dv/v in SubMachine thus serves to extract the de facto reference model.

The user can also control a plot's map projection (Hammer, Mollweide, Robinson, cylindrical equal‐area, orthographic or equidistant cylindrical), color palette (many choices including perceptually uniform color scales), and color mapping (linear versus logarithmic; continuous versus discrete color increments).

SubMachine currently contains two tools for the statistical analysis of tomographic models: “Velocity histograms” (of a horizontal depth slice), and “Velocity‐Depth profiles,” computed over many such slices. Figure 3 shows examples of both tools, for several models. The velocity histograms compute how much map area falls into a given dv/v bin. This is not equivalent to the number of latitude‐longitude pixels per dv/v bin because the area of the regularly interpolated 0.5° × 0.5° pixels varies with latitude (smaller at the poles compared to the equator). Our histogram computation corrects for this.

Figure 4 shows SubMachine's output to a user request for an east‐west cross section through the Iceland hotspot, for 6 *P* wave and *S* wave models. It demonstrates that most current tomography models show low‐velocity material (presumably hot upwelling) in a continuous connection from the core‐mantle boundary to the surface. They also agree on many details of its geometry, which is tilted rather than a straight vertical plume conduit.

The engine of the “Tomography‐Cross‐sections” page is written in Python. Python VTK (Visualization Toolkit) libraries (Quammen, 2015; Schroeder et al., 2004) are employed to efficiently read and slice the 3‐D models. Standard Python plotting libraries are used to set the color bar and to plot the map inset showing the surface projection of the slice.

### 2.2 Tomography Vote Maps

**Figure 5:**
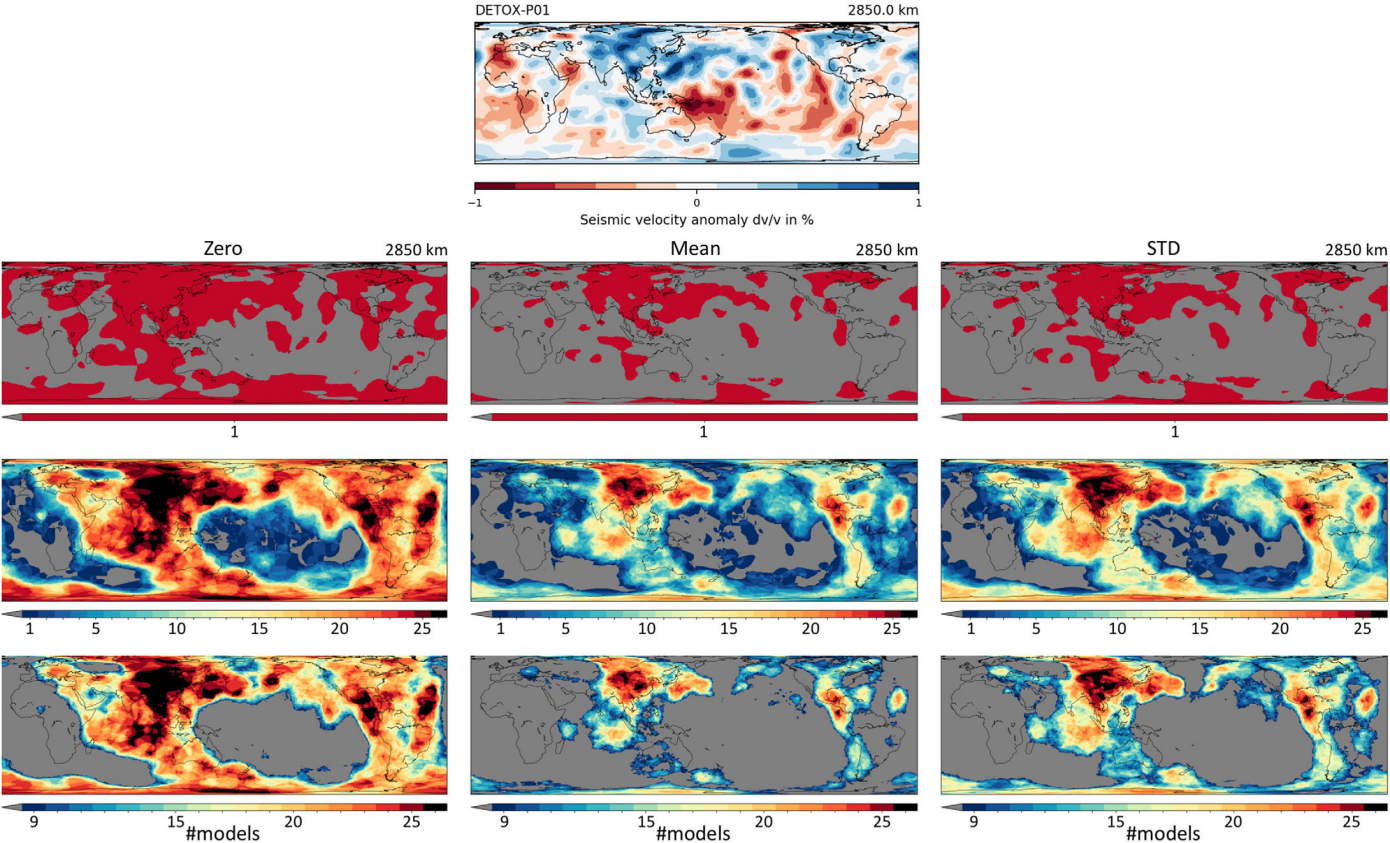
Workflow for the computation of tomography vote maps, on the example of 26 global *P* wave and *S* wave models at 2,850 km depth. (top) One such model. Second row shows the binary spatial masks of “fast” (value 1, red) versus “not fast” votes (value 0, grey) for this specific model. The three columns differ in their threshold criteria: “zero” includes any region dv/v > 0; the stricter “mean” threshold includes only regions dv/v > v_0_, where v_0_ is the average value of all occurrences of dv/v > 0. Similarly, “std” includes only regions of dv/v > v_1_, where v_1_ is the standard deviation of the dv/v > 0 histogram. Preprocessing of individual models consisted of homogenization of the reference velocity model (PREM; Dziewonski & Anderson, 1981) and velocity bias removal. Third row shows plotting output of SubMachine's “Vote Maps” page for these three vote maps, each resulting from the addition of 26 binary masks. Fourth row shows the same vote maps using a stricter color threshold: only areas for which more than nine models estimate fast structure are shaded nongrey. Fast seismic anomalies in the lowermost mantle are thought to represent subducted lithosphere. Hence, these vote maps imply paleo‐subduction zones that roughly coincide with the present and paleo circum‐Pacific (Panthalassa) subduction belt. Better model agreement in the northern than in the southern hemisphere could be due to generally lower imaging resolution in the southern hemisphere, or could reflect an actual relative lack of subducted lithosphere.

Tomography vote maps stack several tomography models in order to reveal regions of agreement and disagreement. This has previously been achieved via *k*‐means cluster analysis, with a focus on slow‐velocity regions (Cottaar & Lekić, 2016; Lekić et al., 2012), and for high‐velocity regions by Shephard et al. (2017), whose workflow we adopt here, extending it to low‐velocity anomalies, the entire mantle column, and more than 30 models (instead of 14). The general workflow is depicted in Figure 5 and is summed up as follows:

A vote map is based on depth slices of *N* tomography models (as in Figure 2), where *N* ≥ 1. A binary threshold of 0 or 1 is applied to every lat‐lon pixel of every depth slice, followed by pixel‐wise summation of all *N* depth slices into a vote map (Shephard et al., 2017). The pixels of a vote map hence take values between 0 and *N*, because each tomography model contributes one “vote” on every pixel. The binary threshold metric for a high‐velocity/low‐velocity vote map is designed to associate the value of 1 (“yes”) with a pixel if its seismic velocity is found to be faster/slower than ambient mantle. Five implemented threshold metrics (“zero,” “mean,” “std,” “rms,” and “median”) permit to choose a lower or higher bar for what “confidently” means. In the example of a high‐velocity vote map, the “zero” metric includes all areas that are seismically fast in that depth slice (dv/v > 0). The stricter “mean” metric includes only regions of dv/v > v_0_, where v_0_ is the average value of all occurrences of dv/v > 0. Similarly, “std,” “rms,” or “median” include only regions of dv/v > v_1_, where v_1_ is the standard deviation, root mean square or median of a model's dv/v histogram at that depth, respectively. Figure 5 illustrates the effects of three threshold metrics (zero, mean and std) on the resulting vote maps.

**Figure 6:**
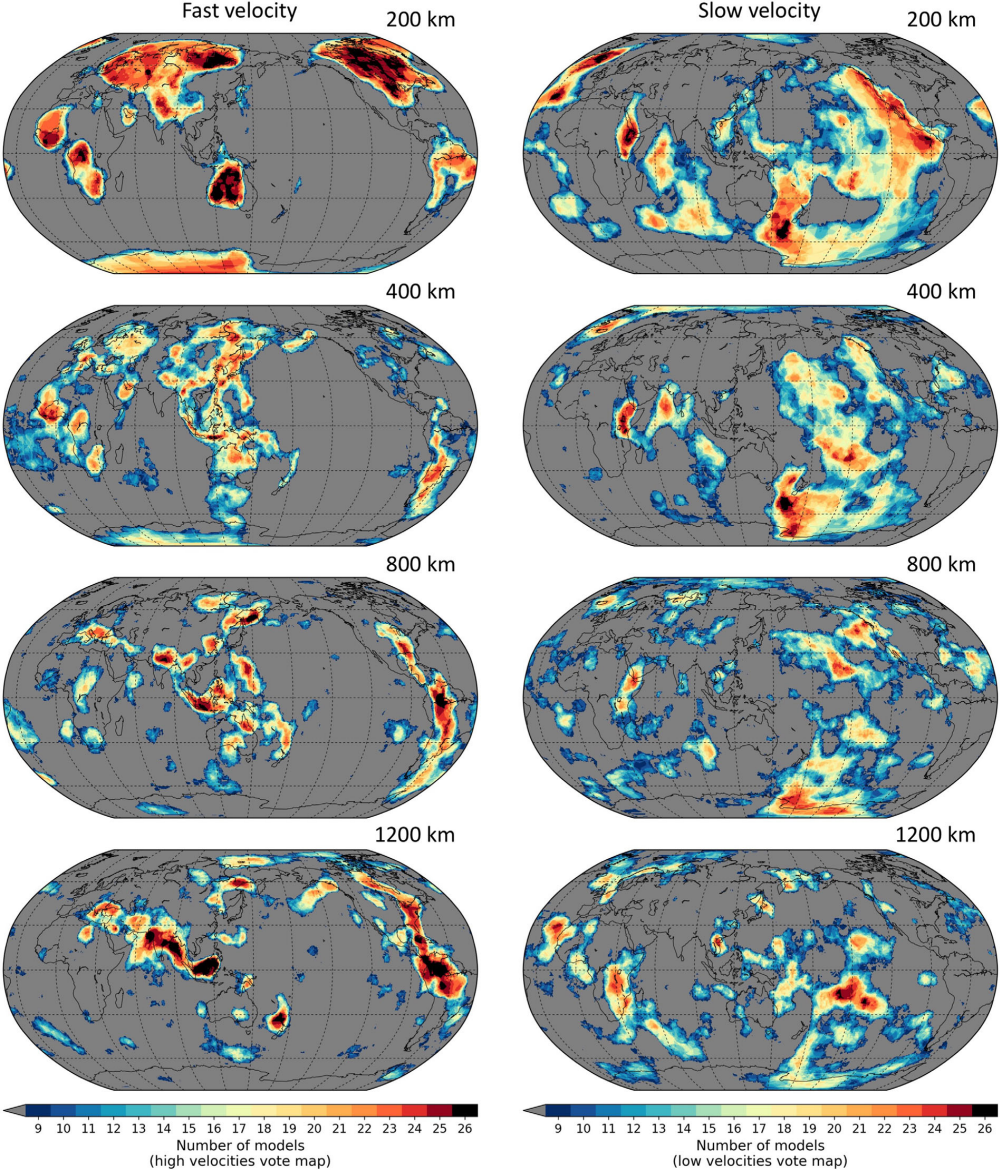
Tomography vote maps from 200 to 1,200 km depth, for (left column) fast‐velocity regions and (right column) slow‐velocity regions. Generated from 26 models as described in the caption of Figure 5 (dv/v bias removed at every depth level for each model; vote threshold criterion is “std”). Longitude, latitude, and vote map scores contained in each 2‐D map slice can be downloaded in ASCII format using the “Download grid” option.

**Figure 7:**
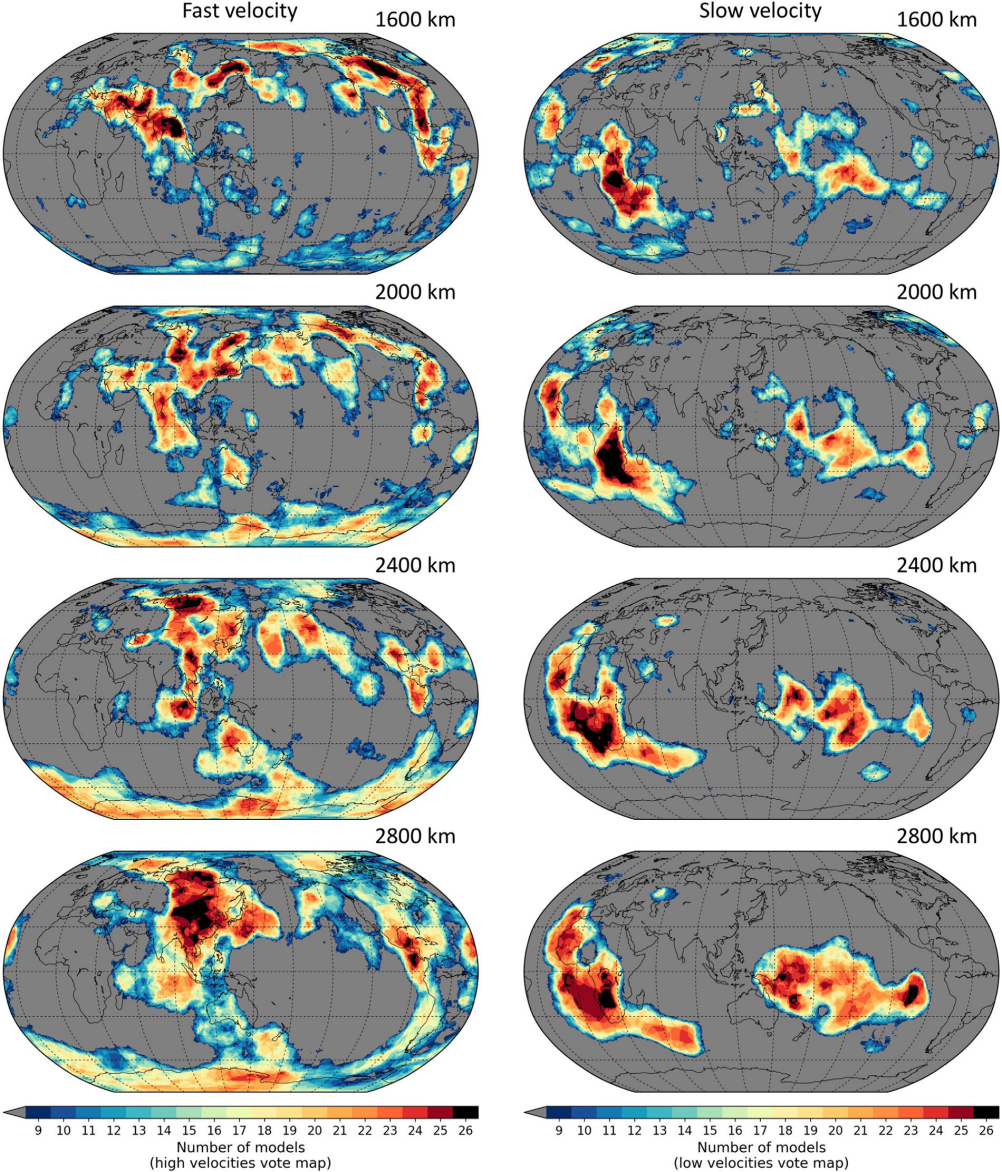
Fast‐velocity (left column) and slow‐velocity (right column) vote maps at 1,600–2,800 km depth. Otherwise as Figure 6.

Regions with higher vote counts indicate stronger agreement across models about the presence of anomalous mantle (dv/v > 0 in the case of a high‐velocity vote map; dv/v < 0 for a low‐velocity vote map). Figures 6 and 7 show examples of vote maps generated from 26 tomography models in the upper and lower half of the mantle, respectively. In the interest of showcasing SubMachine's capabilities, these figures include essentially all available models—but we (highly) recommend a judicial subselection of models.

The rationale for computing vote maps is to increase the confidence in tomographically imaged structure by “polling” different models that are at least partially decorrelated due to using different methods or data. Since there is a large overlap in earthquake locations and seismic stations across different global‐scale inversions, the resulting models will necessarily be correlated to some extent, and hence artifacts in vote maps need not average out. Regions of high vote count do not automatically mean that an anomaly is actually present in the Earth, and low vote counts do not automatically mean that an anomaly is absent.

In more favourable situations, it is possible to average tomography models that were computed from more decorrelated data sets. The prime example is body wave versus surface‐wave models in the upper 300 km of the mantle. Although a “true” artifact is likely to be present only in a subset of models and thus not rise above a moderately low count, the judicial choice of constituent models for a vote map remains the user's responsibility—as uncorrelated as possible, e.g., by including models made from different types of data. Areas of moderate vote count invite further scrutiny regarding the kinds of artifacts typically produced by different imaging methods. This requires studying the original publications of a vote map's constituent models, including resolution tests and other measures of model uncertainty, where provided.

## 3 Complementary Data Sets

### 3.1 Tectonic Plate Reconstructions

To facilitate the linking of mantle structure with plate motion histories, SubMachine provides the functionality to overlay reconstructed plate boundaries (subduction zones, ridges, and transform boundaries) and/or coastlines on seismic tomography models and vote maps. These reconstructions present different, relative and absolute plate motion histories, and their corresponding publications are listed in Table 2. Comparisons between mantle structure and plate reconstructions have broad applications. The geometry of subducted lithosphere should be reconcilable with reconstructed positions of the paleo‐trenches through which the seafloor entered the mantle. For example, Sigloch and Mihalynuk (2013, 2017) proposed a major revision of the Mesozoic paleogeography of western North America, by suggesting that the prevailing assumption of a margin‐hugging (Farallon) trench since 200 Ma is inconsistent with slab geometries under North America. Similarly, Domeier et al. (2017) suggested that an intraoceanic subduction zone traversed the Pacific in the Cretaceous‐Paleocene partly on the basis of subducted lithosphere identified in the midmantle. Working globally, van der Meer et al. (2010) argued that absolute paleolongitude can be determined from a comparison between mantle structure and plate reconstructions, proposing a so‐called subduction reference frame. There are similar links between mantle plumes, as imaged by tomography, and volcanic tracks on tectonic plates, and between the current and former locations of spreading ridges and the slow mantle anomalies imaged beneath them.

**Table 2:** Plate Reconstruction Models Currently Supported in SubMachine

Model	Timeframe (Ma)	Coastlines	Plate boundaries	Spatial extent	Notes	Absolute reference frame
Seton et al. (2012)	200‐0	Yes	Yes	Global	Based on Müller et al. (2008)	0–100 Ma: O'Neill et al. (2005) 110–200 Ma: Steinberger and Torsvik (2008) 83.5–140 Ma, Pacific basin plates: Wessel and Kroenke (2008), Wessel et al. (2006)
Matthews et al. (2016)	410‐0	Yes	Yes	Global	Based on the models of Müller et al. (2016) (0–230 Ma), and Domeier and Torsvik (2014) (250–410 Ma) and regional refinements contained therein	0–70 Ma: Torsvik et al. (2008) 100–230 Ma: Torsvik et al. (2012) 250–410 Ma: Domeier and Torsvik (2014) 83–140 Ma: Wessel and Kroenke (2008)
Zahirovic et al. (2016)	230‐0	Yes	Yes	Global (Tethys focus)	Regional model for the Tethys and Southeast Asia is embedded in an update to the global model of Müller et al. (2016)	0–70 Ma: Torsvik et al. (2008) 105–200 Ma: Steinberger and Torsvik (2008) modified with a 10° longitudinal shift. 83–140 Ma, Pacific basin plates: Wessel and Kroenke (2008)

**Figure 8:**
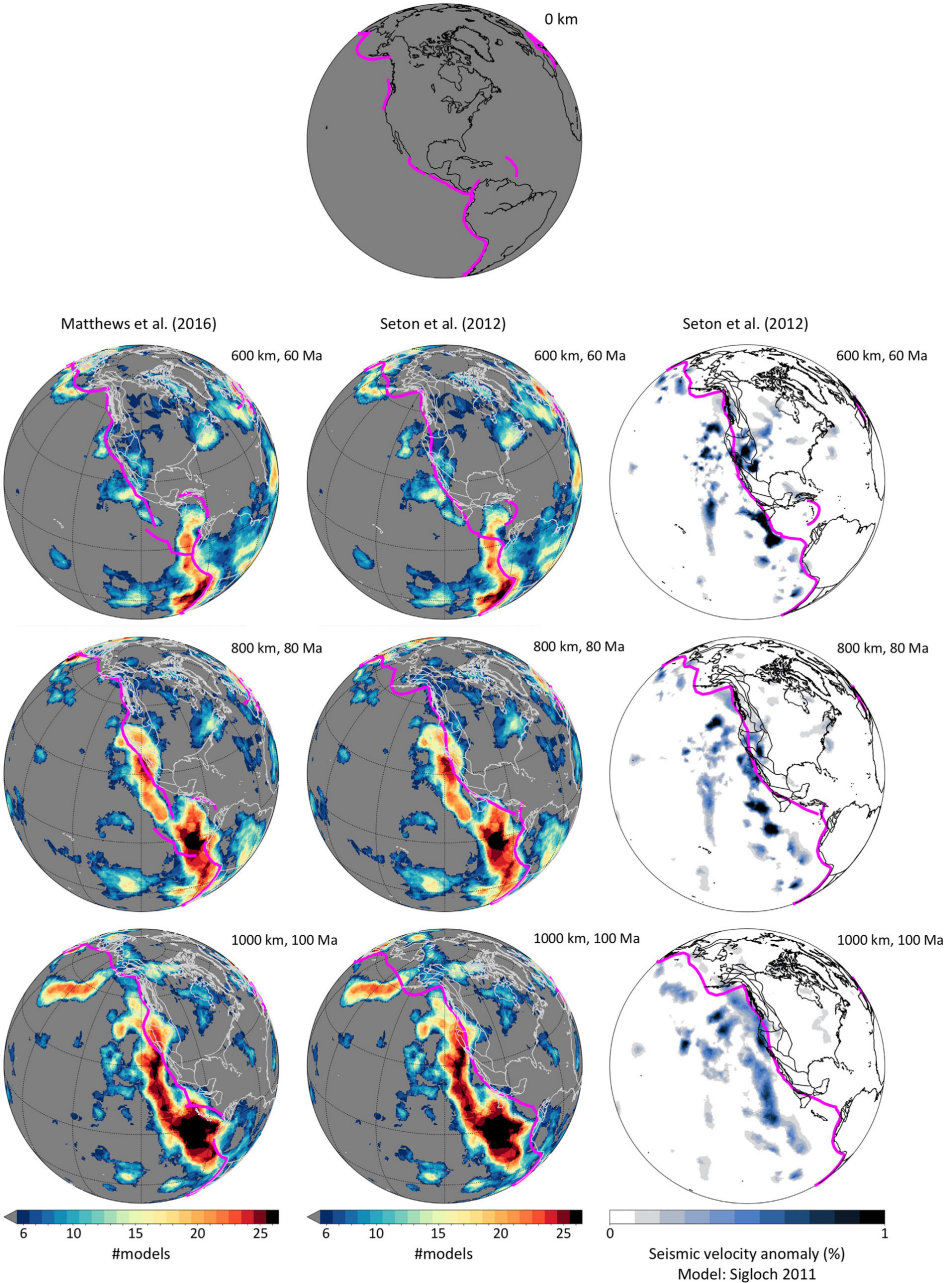
SubMachine's “Tomography‐Depth slices” and “Vote Maps” engines implement the superposition of plate tectonic reconstructions (subduction zones, ridges, transform boundaries, and coastlines) on subsurface maps. This example compares plotting output for two of three supported plate models (magenta subduction zones and white reconstructed coastlines), superimposed on fast mantle structure at 600, 800, and 1,000 km depth. In the left and middle column, these are fast‐velocity vote maps of 26 global tomographies, versus fast anomalies in a regional *P* wave model for North America in the right column (Sigloch, 2011). A depth/time mapping of 10 mm/yr (10 km/myr) is used, which implements the hypothesis that slabs now located at 600/800/1,000 km depth entered the mantle at 60/80/100 million years ago. This enables a comparison of paleo‐trench geometries suggested by subducted slabs to those suggested by plate reconstructions, which are based on surface observations. Plate reconstructions are described in Table 2. Vote maps are generated as in Figure 6, except that areas of less than six votes are shaded uniformly grey.

This initial release of SubMachine implements two kinds of mapping functions between depth in a tomographic model and time in a plate reconstruction. For any given depth slice, the user can manually specify a time for the superimposed plate reconstruction. Alternatively, SubMachine can calculate the reconstruction time automatically according to a user‐specified sinking rate (for a subducting slab). This can either be a single rate, if slab sinking is assumed to be uniform throughout the mantle, or two separate rates for the upper and lower mantle. Recent literature has proposed whole‐mantle slab‐sinking rates between ∼10 and 20 mm/yr (Butterworth et al., 2014; Domeier et al., 2016; Sigloch & Mihalynuk, 2013; Steinberger et al., 2012; van der Meer et al., 2010). In a more granular accounting, slab‐sinking rates are very likely not linear throughout the mantle column, and a particularly prominent change is expected to occur across the transition zone. Different sinking rates may therefore be applicable to different geographic regions or mantle depths, and users may wish to explore a range of sinking rates for any given time‐depth comparison. Several examples of SubMachine's depth‐time mapping capabilities are shown in Figure 8.

### 3.2 Geodesy: Geoid, Gravity and Topography

A geoid surface is an equipotential surface of gravitational potential energy. Global‐scale geoid observations, mainly from satellites, are used in many geodetic, oceanographic and geophysical applications, and can serve to constrain subsurface structure in mantle convection models or joint seismic‐geoid tomographies (Simmons et al., 2009). The smooth but irregular shape of the geoid is due to the uneven distribution of mass within and on the surface of the solid Earth. A positive gravity anomaly is caused by a mass excess and results in a geoid high relative to the reference ellipsoid. A viscous mantle complicates the picture, because the secondary effects of dynamic topography (at the surface, the core‐mantle boundary, and due to deflections of discontinuities in the transition zone) tend to affect the geoid in the opposite sense than that of the density anomalies driving mantle flow (Hager, 1984). For example, a dense sinking sphere causes a geoid high, but the surface depression in its wake, which is due to viscous downward drag, causes a geoid low. Due to poorly known mantle rheologies, the sensitivity of the geoid to the analogous effects of indented discontinuities at “410 km,” “660 km,” and the core‐mantle boundary is less certain. The causal relationship between subduction, the geoid and deep Earth structure motivates the joint consideration of geoid observations, seismologically imaged mantle anomalies and plate reconstructions.

Satellite‐based measurements of changes in the Earth's gravitational field over the marine environment, which are related to density variations below the oceans, can be detected via changes in the ocean surface. Based on radar altimetry data, such measurements of marine gravity and bathymetry have been used to generate increasingly high‐resolution images of the seafloor topography and related subsurface tectonic structures (e.g., Sandwell et al., 2014). Marine gravity, along with its high‐pass derivatives such as vertical gravity gradients (VGGs), can image large‐scale features such as sedimentary basins and mid‐ocean ridges, as well as smaller wavelength features including seamounts and fracture zones.

**Figure 9:**
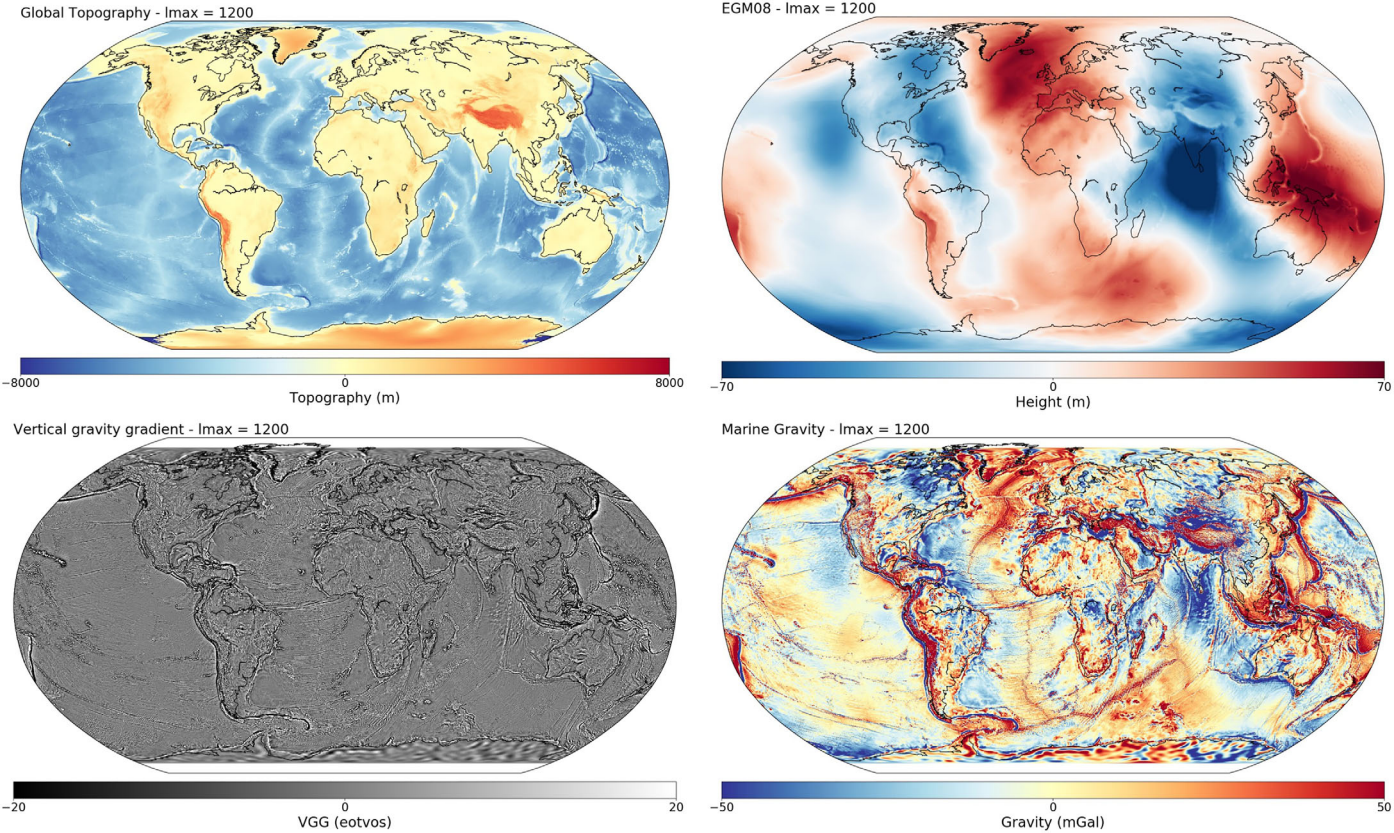
SubMachine visualizes selected global surface data sets that may inform the study of subsurface structure. Global topography, geoid, vertical gravity gradient and marine gravity can be visualized to user‐specified spatial resolution (spherical harmonic degrees of 4 ≤ lmax ≤ 1,200). Color bars and map projection are customizable.

The “Geodesy” functionality of SubMachine can render global topography (Olson et al., 2014), geoid (Pavlis et al., 2012), marine free‐air gravity and vertical gravity gradient (Sandwell et al., 2014) maps, as shown in Figure 9. Based on the user‐specified lmax, SubMachine plots a model for all spherical harmonic degrees up to and including lmax. Geodetic data sets can currently only be plotted in separate figures, not superimposed on tomography slices or vote maps.

### 3.3 Global Crustal Structure

CRUST1.0 (Laske et al., 2013) is a global crustal model specified on a 1° × 1° grid. In each 1° cell, boundary depth, compressional velocity (Vp), shear velocity (Vs) and density are given for eight layers: water, ice, sediment layers (upper, middle, and lower), and crystalline crust (upper, middle, and lower). Bathymetry, topography, and ice thickness are derived by binning and averaging ETOPO1 data (Amante & Eakins, 2009), a 1 arc min model of the Earth's global relief. Moho depth in CRUST1.0 is based on 1° averages of crustal thickness data sets from active source seismic studies, from receiver function studies and published Moho maps, as well as estimated thicknesses using gravity constraints (Laske et al., 2013).

**Figure 10:**
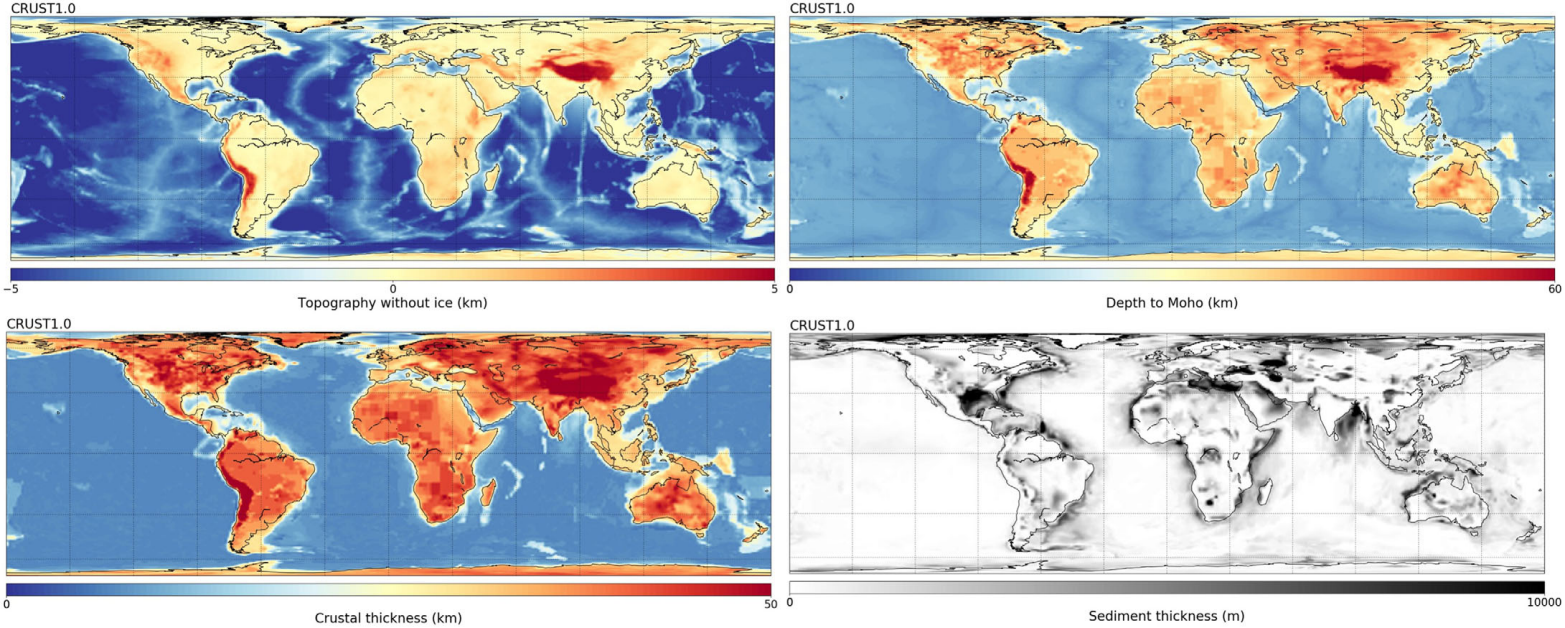
SubMachine's “Crust 1.0” functionality visualizes crustal model CRUST1.0 (Laske et al., 2013). This includes topography, depth to Moho, crustal thickness, and sediment thickness, as shown here. Compressional velocity, shear velocity, density, and thickness of individual layers can be plotted as well. The CRUST1.0 model defines eight layers; see text for details. Map projection and color bar are customizable.

The “Crust 1.0” tab of SubMachine can visualize all these parameters on different map projections and color bars (Figure 10).

### 3.4 Normal Mode Observations

Normal modes, the solid Earth's free oscillations, are frequency‐split due to 3‐D heterogeneities that break the spherical symmetry. Some of this frequency splitting is caused by the same mantle heterogeneities that are imaged by body and surface‐wave tomography models. Hence, it is pertinent to compare to normal mode splitting observations, which are visualized as splitting function maps. These maps represent the local radial average (depending on the sensitivity kernel) of the underlying heterogeneity sampled by a particular normal mode below each point.

**Figure 11:**
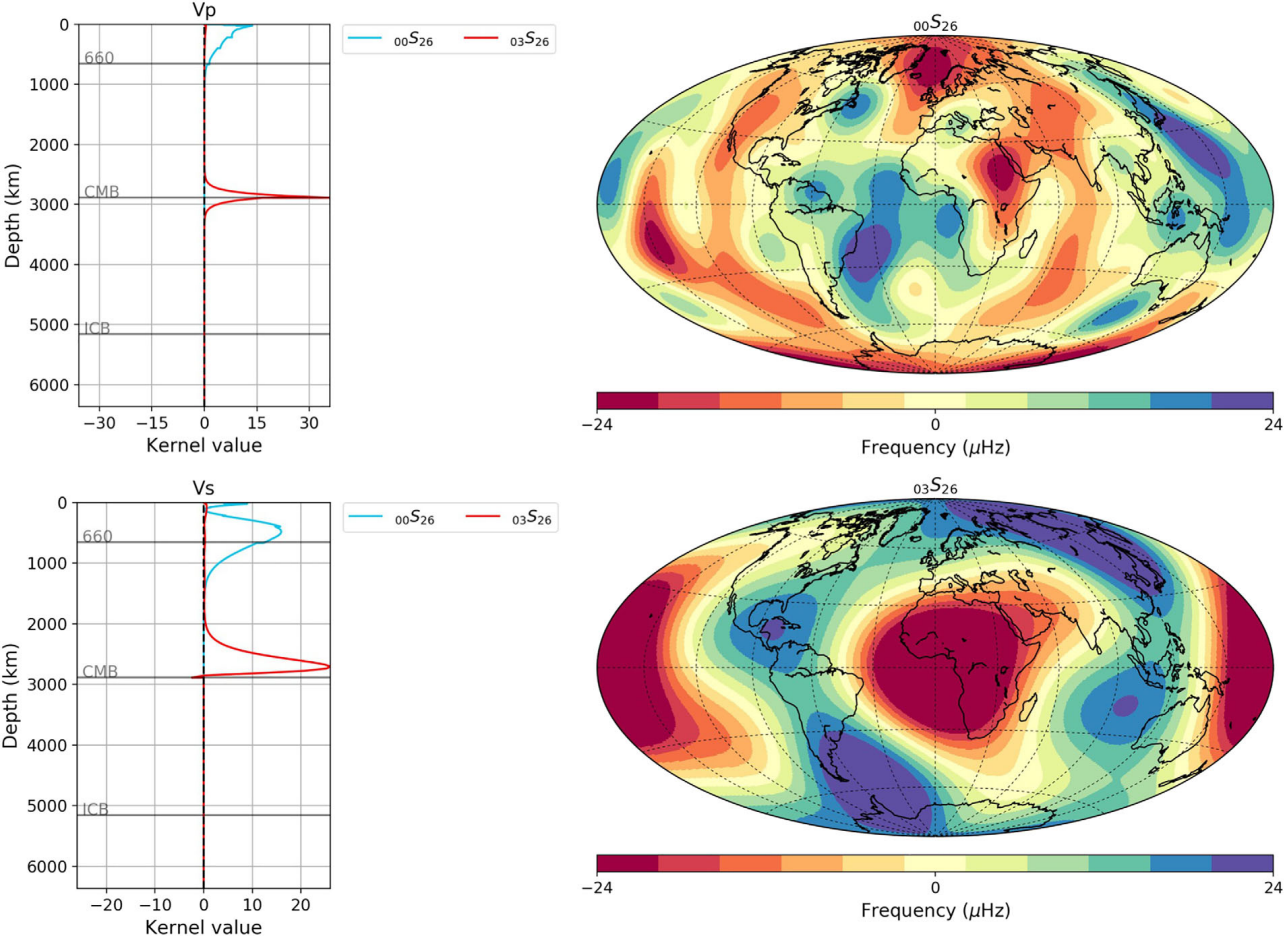
Output of SubMachine's “Normal Modes” functionality, plotting (right) two normal mode observations and (left) their V_P_ and V_S_ sensitivity kernels as a function of depth. Mode _00_S_26_ is seen to be mainly sensitive to upper mantle structure (cyan lines), mode _03_S_26_ mainly to lowermost mantle structure (red lines). Nineteen modes are currently supported (Koelemeijer, 2014; Koelemeijer et al., 2013). Map projection, color bar, and reference model are customizable.

A “Normal Modes” tab has been implemented in SubMachine for visualizing normal modes and their associated sensitivity kernels (Vs, Vp, and density ρ). Currently, 19 normal modes (_00_S_21–30_, _01_S_11–14_, _02_S_15–17_, _02_S_25_, and _03_S_26_) are supported, based on Koelemeijer et al. (2013) and Koelemeijer (2014). These modes are divided into two groups based on the depth extent of their sensitivity kernels: upper mantle and lower mantle sensitive modes. In the latter, nine core‐mantle boundary Stoneley modes _1_S_11_, _1_S_12_, _1_S_13_, _1_S_14_, _2_S_15_, _2_S_16_, _2_S_17_, _2_S_25_, and _3_S_26_ are included which are a unique class of normal modes with extremely focused sensitivities to wave speed and density variations in the D″ region. Figure 11 shows output of the “Normal Modes” tab of two mode observations with their associated V_P_ and V_S_ sensitivity kernels as functions of depth.

## 4 Discussion

### 4.1 Pros and Cons of Web‐Based Visualization

There are several advantages to web‐based visualization tools. No software installation is required of the user, except for a web browser connected to the internet. Hence, there are no platform restrictions or update requirements either. All processing and visualization steps are executed on a server, requiring no programing by the user. The results and outputs can be easily reproduced and shared without transferring actual files because each user request to the SubMachine portal generates a unique URL (plain text string) that can be saved and shared.

Disadvantages of web portals include access limitations when the website is under maintenance or when the server goes down. The speed of computations on a server, although acceptable, is somewhat decreased compared to local machines.

SubMachine and other tomography portals (e.g., Becker & Boschi, 2002; Hutko et al., 2017; Kim et al., 2016) render 2‐D slices extracted from volumetric data sets. As an extension to 3‐D rendering, isosurface (2‐D hyperplanes) or true volume rendering are highly desirable visualization options but are computationally and technically challenging. While becoming more common, 3‐D rendering is still not routine even in tomography research groups. Mantle heterogeneities are much less structured and intuitive to grasp than, for example, medical tomography images. From extensive experimentation (Hosseini, 2016; Hosseini et al., 2014; Sigloch, 2011; Sigloch et al., 2008), we have found it necessary to build elaborate and customized workflows in scripting environments such as Matlab and ParaView (through Python). Interfacing those programs with a web‐based GUI is even more challenging, technologically and in terms of user experience. These workflows require the customization of numerous parameters—related to scene masking, coloring, choice of 3‐D renderer, viewing angles and lighting—in order to make a mantle‐scape digestible to the brain. For the next release of SubMachine, we are investigating the usefulness of implementing a few standard 3‐D views with a limited number of parameters, such as map views of isosurfaces. Some examples of 3‐D rendered tomography models and vote maps are collected in “Posters/Videos” tab of the SubMachine site.

### 4.2 Quantitative and Qualitative Comparison of Earth Models

SubMachine supports both quantitative and qualitative comparisons of tomography models in various ways. It offers instant side‐by‐side comparison of any slice through any number of tomography models, in a uniform, customizable format. The raw dv/v values underlying the plots can be downloaded and processed by the user, in order to visually or computationally highlight features of interest. Tomography models can be queried and compared statistically via the histograms and velocity‐depth profiles. Vote maps can be constructed by combining two or more tomography models. In particular, vote maps for *N* = 2 models take the value 1 in areas where the two models disagree (on either fast or slow structure), and the values 0 or 2 in areas where they agree.

By contrast, model comparisons in the literature typically feature raw velocity data from only a few, readily accessible models, or resort to the qualitative comparison of graphics reproduced from original publications, which often use different reference models, section locations, and color schemes.

### 4.3 Outlook: Additional Types of Earth Models and Assessment of Uncertainties

While the collection is currently focused on relatively recent models, it is desirable to collect the raw data of older models before they become unavailable, especially historically influential ones. For anisotropic *S* wave models, only the visualization of the isotropic (Voigt) average is currently supported, but we plan to extend this to the visualization of anisotropy. Depending on community interest and contributions, SubMachine could also host and serve global‐scale magnetotelluric models, or outputs of mantle convection simulations. Work is underway on adding new functionalities, including the superimposition of seismic event locations retrieved from various catalogues (Hosseini & Sigloch, 2017) and shear wave splitting measurements (Wüstefeld et al., 2009) on tomography models.

Another line of development will focus on more direct means for assessing model uncertainties. This would include resolution test inputs and outputs for hosted models; summary statistics of measurements sensitivities (e.g., column density sums of the sensitivity matrix or raypath coverage); model sequences along an L‐curve (instead of only the preferred, final model), which permits artifacts to be identified more readily; or entire resolution matrices, if available (Ritsema et al., 2011, 2007, 1999). This will require a higher degree of community input because such uncertainty tests (which are expensive to compute and produce volumetrically larger outputs than the models themselves) are currently not archived and distributed to nearly the same extent as the final, preferred models.

## 5 Conclusions

We have presented and discussed the initial release of the SubMachine portal, a collection of web‐based tools for the interactive visualization, analysis, and quantitative comparison of global‐scale data sets of the subsurface. Its main functionalities and data sets were described through examples, such as the visualization and analysis of seismic tomography models, the generation of vote maps by combining several tomography models, visualization of additional data sets including geoid, topography, marine gravity, vertical gravity gradient, global crustal structure, and normal mode observations. Mantle structure can be linked with plate motion histories by overlaying surface reconstructions of paleo‐plate boundaries on depth slices from seismic tomography. Various ways for comparing tomography models were discussed, e.g., side‐by‐side comparison of any slice through any number of tomography models, in a uniform, customizable format; quantitative model comparison via the histograms and velocity‐depth profiles; model comparison by creating vote maps.

We welcome community input on features and model contributions. Source code is in a GitHub repository and can be made available upon request. The tomography models in their raw form (large files of dv/v values in their original format, our homogenized format or VTK format) can be made available upon request and where we have their authors' permission to share them.
